# Improving Na_2_Ti_3_O_7_ Anode Performance in Sodium-Ion Batteries via a Al Doping

**DOI:** 10.3390/nano15120885

**Published:** 2025-06-08

**Authors:** Chen Wu, Yuandong Xia, Kejing Song, Yongda Cao, Chenzhi Huang, Jiayi Chen, Yuan Wang, Chunliu Xu

**Affiliations:** 1 Natural Gas Research Institute, PetroChina Southwest Oil & Gasfield Company, Chengdu 610213, China; cwu@petrochina.com.cn (C.W.); songkejing@petrochina.com.cn (K.S.); caoyongda@petrochina.com.cn (Y.C.); huang_chzh@petrochina.com.cn (C.H.); 2 College of Chemical Engineering, Sichuan University, Chengdu 610065, China; 2024141490094@stu.scu.edu.cn (Y.X.); chen_jiayiii@163.com (J.C.); 3 Research Institute of Frontier Science, Key Laboratory of Advanced Technologies of Materials-(Ministry of Education), School of Materials Science and Engineering, Southwest Jiaotong University, Chengdu 610031, China

**Keywords:** sodium-ion batteries, Na_2_Ti_3_O_7_, Al doping, electrochemical performance, Coulombic efficiency

## Abstract

Na_2_Ti_3_O_7_ (NTO), with low sodium insertion potential (~0.3 V vs. Na^+^/Na) and potential for high-energy-density batteries, is regarded as one of the most promising anode materials for sodium-ion batteries (SIBs). However, its practical application is hindered by poor electronic conductivity, sluggish Na⁺ (de)intercalation kinetics, and interfacial instability, leading to inferior cycling stability, low initial Coulombic efficiency, and poor rate capability. In this work, micron-sized rod-like NTO and Al-doped NTO (NTO-Al) samples were synthesized via a one-step high-temperature solid-state method. Al doping slightly reduced the size of NTO microrods while introducing oxygen vacancies and generating Ti^3+^, thereby enhancing electronic conductivity and reducing ionic diffusion resistance. H_2_-TPR confirms that doping activates lattice oxygen and promotes its participation in the reaction. The optimized NTO-Al0.03 electrode delivered a significantly improved initial charge capacity of 147.4 mA h g^−1^ at 0.5 C, surpassing pristine NTO (124.7 mA h g^−1^). Moreover, it exhibited the best cycling stability (49.5% capacity retention after 100 cycles) and rate performance (36.3 mA h g^−1^ at 2 C).

## 1. Introduction

Insertion-based electrodes are widely preferred in battery systems because alkali metal ions can reversibly intercalate into their layered structures without inducing significant crystallographic damage, ensuring long-term cycling stability [1,2]. The superior electrochemical behavior of insertion-type electrodes can be attributed to intercalation pseudocapacitance, where capacity retention remains stable across a wide range of current densities [3]. As a result, such electrodes typically exhibit outstanding cycling durability and high-rate performance. However, the selection of suitable anode materials for insertion-type sodium-ion batteries (SIBs) remains limited. Unlike in lithium-ion batteries (LIBs), many high-rate electrode materials fail to deliver comparable performance in SIBs due to the larger ionic radius of Na^+^, which hinders efficient ion transport and storage [4].

Na_2_Ti_3_O_7_ (NTO) is a potential insertion-type anode for sodium-ion batteries, as its layered TiO_6_ octahedral framework enables reversible Na^+^ intercalation with a theoretical capacity of 178 mAh g^−1^ [5,6]. Notably, NTO exhibits a low redox plateau at 0.3 V, representing the first oxide-based material capable of sodium storage at such a low potential [7]. However, its poor electronic conductivity restricts achievable capacity, falling short of theoretical values and underperforming compared to hard carbons or organic anodes [8,9]. Furthermore, rapid capacity decay during prolonged cycling hinders its practical viability in SIBs [10]. Therefore, improving the intrinsic conductivity of NTO remains a critical challenge for advancing its electrochemical performance.

The design of nanostructure, doping, and nanocomposites were normally strategies to address the above challenges [11,12,13]. Among them, the introduction of defects or dopant ions into the crystal structure enables precise control over both bulk and surface architectures [14], thereby enhancing the intrinsic ionic and electronic conductivity of active materials [15,16,17,18,19]. This structural modification facilitates more efficient and rapid sodium-ion storage. It is reported that n-type dopants can enhance conductivity by providing excess electrons and partially reducing Ti^4+^ to Ti^4+^ [20]. When p-type lanthanide doping is employed, oxygen vacancies are readily introduced, thereby improving the electrochemical performance of NTO [21]. As demonstrated by Pak et al., the presence of defect structures can reduce the activation energy for Na^+^ migration along the 1D [010] pathway within the interlayer space to below 0.4 eV [22]. In the TiO_2_ production process, titanyl sulfate (TiOSO₄) forms as an intermediate product, which undergoes hydrolysis to yield metatitanic acid. The industrial-grade TiOSO_4_ solution pre-hydrolysis typically contains multiple impurity ions, such as Ca, Mg, Al, and Si [23,24]. Given the cost-effectiveness considerations for NTO doping, investigating these naturally occurring impurity ions from upstream precursors for NTO modification represents a promising strategy.

Inspired by the above, Al^3+^ (0.535 Å) and Ca^3+^ (1.00 Å,) were selected as dopants for Ti^4+^ sites (0.605 Å) based on their ionic radii and anticipated defect chemistry. The smaller Al^3+^ could enable stable substitution at Ti^4+^ sites with reduced lattice distortion, while the larger Ca^2+^ induces controlled lattice expansion to facilitate Na^+^ diffusion. In this work, a series of Al- and Ca-doped NTO electrode materials (NTO-Al and NTO-Ca) were first synthesized, with their cycling stability and rate capability systematically evaluated. The results demonstrated superior performance enhancement from Al doping compared to Ca doping. The Al-doped NTO was then selected for in-depth investigation, where XRD, TEM, and XPS characterizations revealed its modified lattice structure, optimized morphology, and altered surface chemistry. Further mechanistic studies through dQ/dV curve analysis, GITT measurements, and EIS testing provided critical insights into the improved sodium-ion diffusion kinetics and enhanced charge transfer properties induced by Al doping.

## 2. Materials and Methods

### 2.1. Materials

Anatase TiO_2_ (99.0%) and sodium carbonate (Na_2_CO_3_, ≥99.0%, AR grade) were purchased from Sinopharm Chemical Reagent Co., Ltd. (Shanghai, China). Aluminum oxide (Al_2_O_3_, ≥99.99%) was purchased from Kelong Chemical Reagent Company (Chengdu, China). Anhydrous ethanol (C_2_H_5_OH, AR grade) was purchased from Chengdu Jinshan Chemical Reagent Co., Ltd. (Chengdu, China). Polyvinylidene fluoride (PVDF, battery grade) was purchased from Taiyuan Lizhiyuan Battery Materials (Taiyuan, China). N-Methylpyrrolidone (NMP, ≥99.0%, AR grade) and Super P (battery grade) were purchased from Guangdong Zhuguang New Energy Technology Co., Ltd. (Guangzhou, China). Carboxymethyl cellulose (CMC, AR grade) was purchased from Aladdin Reagent Co., Ltd. (Shanghai, China). Styrene-butadiene rubber (SBR, AR grade) was purchased from Shanghai Huiping Chemical Co., Ltd. (Shanghai, China). Dimethyl carbonate (DMC, AR grade), NC-009 electrolyte (NaClO_4_/EC+DEC (1:1 *v*/*v*), battery grade), and NP-035 electrolyte (NaPF_6_/DME (100%), battery grade) were purchased from Suzhou Duoduo Chemical Technology Co., Ltd. (Suzhou, China). Copper foil (9 μm × 170 mm), corundum crucible, and quartz tube were purchased from Guangdong Kelude New Energy Technology Co., Ltd. (Guangzhou, China). Glass fiber (GF/D) was purchased from Whatman Ltd. (Maidstone, UK). Coin cell casings (CR2025) were purchased from Shenzhen Kejing Technology Co., Ltd. (Shenzhen, China). High-purity argon gas (Ar, ≥99.9995%, *v*/*v*) was purchased from Sichuan Jinrui Gas Co., Ltd. (Chendu, China). Deionized water (H_2_O, AR grade) was prepared in-house.

### 2.2. Preparation of NTO, NTO-Al, and NTO-Ca Anode

All NTO and NTO-Al doped samples were synthesized via the identical solid-state reaction method. Typically, 1.02 g of Na_2_CO_3_ and 2.20 g TiO_2_ (with slight Na_2_CO_3_ excess relative to stoichiometric ratio) were manually ground with ~3 mL ethanol for 30 min. For Ca- or Al-doped NTO, predetermined amounts of Al_2_O_3_ or CaCO_3_ were introduced prior to grinding. The homogenized powder mixtures were then calcined in a tube furnace at 900 °C for 15 h under static air, with controlled heating/cooling rates of 5 °C min^−1^. According to previous reports, there are two possible substitution sites in sodium transition metal oxide materials: the transition metal site (Ti site) and the Na site. Based on ionic radius considerations, Al^3+^ (0.53 Å) has a closer ionic radius to Ti^4+^ (0.61 Å), suggesting Al likely occupies Ti sites, while Ca^2+^ (0.99 Å) closely matches Na^+^ (1.02 Å), indicating Ca preferentially substitutes Na sites. Therefore, according to the stoichiometric ratio of guest ions, the Al-doped samples were designated as Na_2_Ti_3-x_Al_x_O_7_ (NTO-Alx, x = 0.01, 0.03, and 0.05), and the Ca-doped samples as Na_2-y_Ca_y_Ti_3_O_7_ (NTO-Cay, y = 0.02, 0.04, 0.06, 0.08, and 0.10).

### 2.3. Preparation of Electrode and Cell

The electrode slurry was prepared by blending the active material (70 wt%), Super P carbon (20 wt%), and PVDF binder (10 wt%) in N-methyl-2-pyrrolidone (NMP) solvent, followed by coating onto copper foil current collectors. The coated electrodes were dried at 90 °C for 12 h prior to cell assembly. The battery assembly was conducted in an argon-filled glovebox with oxygen and water contents maintained below 0.5 ppm. CR2025 coin cell hardware was used for the assembly. The positive electrode shell was first placed flat on the working surface, followed by careful placement of the pre-weighed electrode disk at its exact center. A glass fiber separator was then positioned over the electrode, and an appropriate amount of electrolyte was dispensed. For half-cells, a freshly cut sodium metal disk was used as the counter electrode before sealing the negative shell. The cells were crimped at 0.65 MPa pressure using a hydraulic crimper. After removal from the glovebox, the assembled cells were allowed to stabilize for at least 12 h prior to electrochemical testing.

### 2.4. Electrochemical Performance

Differential capacity (dQ/dV) curves were derived from smoothed GCD data to identify voltage plateaus, with peaks matching those in CV. A wider differentiation interval (k > 1) reduced noise while preserving resolution. Measurements used a Neware BTS-610 system (2.5–0.01 V vs. Na/Na^+^). CV tests (LK 9805 workstation, Tianjin, China) were performed at 0.01–2.5 V (vs. Na^+^/Na) with varying scan rates. The pseudocapacitive contribution was quantified by fitting scan-rate-dependent CV curves. GITT (CT2001A LANHE workstation, Wuhan, China) applied 17.7 mA g^−1^ pulses (10 min) followed by 1 h rests. Diffusion coefficients were calculated from potential relaxation data. All tests were at 25 °C.

### 2.5. Hydrogen Programmed Temperature Reduction (H_2_-TPR)

The experimental procedure was as follows: First, 100 mg of the sample was weighed and placed in a U-shaped quartz tube. The temperature was raised from room temperature to 200 °C at a rate of 10 °C min^−1^ for drying pretreatment. The sample was purged with He gas (50 mL min^−1^) for 1 h, cooled to 50 °C, and then exposed to a 10% H_2_/Ar mixture (50 mL min^−1^) for 0.5 h until baseline stabilization. Subsequently, the temperature was increased to 900 °C at 10 °C min^−1^ under the same gas flow for desorption, while a thermal conductivity detector (TCD) monitored the signals of reduced gases.

### 2.6. Charactrization

A field-emission scanning electron microscope (FE-SEM; JEOL JSM-7500F, Tokyo, Japan) was used to observe the morphology and elemental distribution of the samples after heat treatment. X-ray diffraction (XRD) measurements were performed on a Bruker D8 Advance diffractometer with Cu Kα radiation (λ = 1.5406 Å). Fourier-transform infrared spectroscopy (FTIR) was conducted using a Bruker R200-L spectrometer. Raman spectra were acquired on a Thermo DXRxi instrument with a 532 nm laser. X-ray photoelectron spectroscopy (XPS) was performed on a Thermo Scientific K-Alpha+ spectrometer (Al Kα excitation source). The binding energy scale was calibrated using the C 1s peak (284.8 eV). The microstructure of the samples was examined by transmission electron microscopy (TEM; FEI Talos F200 S). The component of Ti and Al was detected by the inductively coupled plasma-optical emission spectrometer (ICP-OES, Thermo Fisher Scientific Inc., Waltham, MA, USA).

## 3. Results and Discussion

The XRD patterns of all samples were collected as shown in Figure S1. The XRD patterns of NTO and NTO-Alx (Figure S1a) show that all diffraction peaks can be indexed to a monoclinic structure with space group P21/m (PDF card No. 72-0148), confirming the layered NTO crystal structure [25]. The sharp peaks indicate high crystallinity achieved through high-temperature calcination. The strongest peak at ~10.52° corresponds to the (100) plane with an interlayer spacing of 0.84 nm [26]. No impurity peaks (e.g., TiO_2_ or Al_2_O_3_) were detected, suggesting successful Al incorporation into the NTO lattice. The NTO-Cay samples also match well with the NTO pattern (Figure S1b). However, a small amount of Na_2_Ti_6_O_13_ (PDF No. 73-1398) was detected in NTO-Ca0.02 [27]. The entry of Ca into Na sites leads to less Na in the sample, which is consistent with the lower Na content of Na_2_Ti_6_O_13_ compared to Na_2_Ti_3_O_7_ [28].

The cycling performance of all samples was further investigated at 0.5 C for 100 cycles (Figure S2). The related capacity in initial and 100th cycles are displayed in Figure S2c. The Ca-doped samples did not significantly improve the first-cycle reversible capacity of NTO but enhanced cycling stability. Specifically, the reversible capacity after 100 cycles increased from 57.1 mA h g^−1^ for pristine NTO to 70.9 mA h g^−1^ for the optimal Ca-doped sample (NTO-Ca0.06). In contrast, Al doping improved the initial reversible capacity, increasing it from 124.7 mA h g^−1^ (NTO) to 147.4 mA h g^−1^ for the best-performing Al-doped sample (NTO-Al0.03), leading to a higher 100 cycle capacity of 72.9 mA h g^−1^. The related rate performance is shown in Figure S3. Clearly, NTO-Cay electrodes show a relatively low capacity at various current density compared with NTO, which could be ascribed to that Ca doping has a negative impact on the migration barrier of Na. Notably, NTO-Al0.03 delivered a superior rate performance. Thus, the Al-doped samples were selected for further investigation. Compared with the previous reported anode, NTO-Al0.03 delivered a good electrochemical performance [29,30,31].

**Figure 1:**
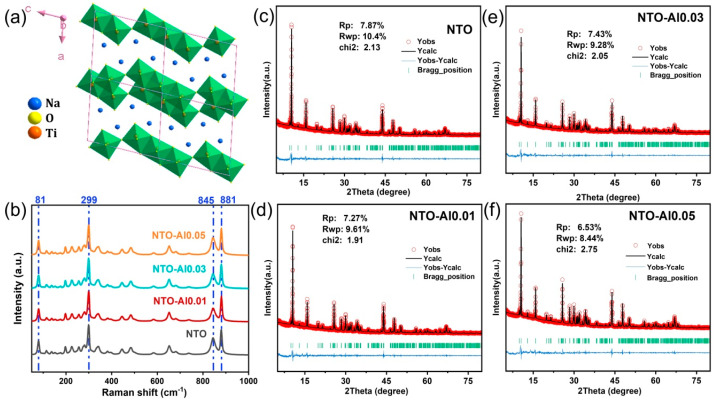
(**a**) Schematic of the crystal structure of Na_2_Ti_3_O_7_, (**b**) Raman spectra of the as-synthesized NTO and Al doping samples, (**c**–**f**) Rietveld refinement of NTO, NTO-Al0.01, NTO-Al0.03, and NTO-Al0.05.

The crystal structure of NTO is illustrated in Figure 1a, where TiO_6_ octahedra (composed of central Ti atoms and surrounding O atoms) form zigzag layers through edge-sharing connections. Na^+^ ions are intercalated between these layers, occupying two distinct sites. While ionic bonds form between Na^+^ ions and TiO_6_ octahedra, the octahedral framework itself maintains strong covalent bonding, constituting the Na_2_Ti_3_O_7_ structure. Notably, identifying the doping sites is crucial for understanding how crystal structure influences the electrochemical performance of NTO anode materials. The incorporation of Al^3+^ into the NTO lattice is particularly important due to the valence differences among Na^+^, Al^3+^, and Ti^4+^. Charge balance considerations suggest that Al substitution at different sites would lead to varying stoichiometric ratios of Al, Na, and Ti. To verify the precise chemical position of Al^3+^ doping, an ICP elemental analysis was performed. Although the Na content could not be accurately determined due to its instability during ICP testing, Table S1 presents the mass percentages of Al and Ti along with their molar ratios. The observed Ti/Al ratio trends align well with theoretical predictions for Ti-site doping, consistent with ionic radius considerations. Figure 1b displays the Raman spectra of different samples. Characteristic peaks were observed at 81 cm^−1^ (Na_1_), 299 cm^−1^ (O_2_-Ti_2_-O_3_ vibration), 845 cm^−1^ (Ti_1_-O_4_ stretching vibration), and 881 cm^−1^ (O_3_-Ti_2_-O_5_ symmetric stretching) [32,33]. Using the strongest peak at 299 cm^−1^ as a reference, Table S2 compares the relative intensities of these prominent peaks. The results show that NTO-Al0.05 maintains vibrational modes most similar to pristine NTO, while NTO-Al0.03 exhibits the most significant reduction in O_3_-Ti_2_-O_5_ symmetric stretching intensity. This phenomenon can be attributed to altered bond energies resulting from Ti-O-Al configurations.

To obtain further insights into the crystal structure of the samples, Rietveld refinement was performed on the XRD patterns [34]. The refinement profiles (Figure 1c–f) present the experimentally observed and calculated difference patterns for four representative samples, with the corresponding refinement results summarized in Table S3. The refinements were conducted using the FullProf software (June 2020 version) suite, and the relatively small values of Rwp and χ^2^ confirm the reliability of the refinement results [35]. Comparative analysis of the unit cell parameters obtained from XRD refinement revealed systematic variations induced by Al doping. The unit cell volume initially decreased and then increased with higher doping concentrations. The initial volume contraction can be attributed to the smaller ionic radius of Al^3+^ (0.53 Å) compared to Ti^4+^ (0.61 Å), while the stronger Al-O bond energy may also contribute to this slight structural shrinkage. The evolution of lattice parameters (a, b, c) showed less obvious trends. For NTO-Al0.01 and NTO-Al0.03, the volume reduction primarily resulted from decreases in the a and c parameters, whereas the subsequent volume expansion in NTO-Al0.05 was also dominated by changes in these same directions. The significant parameter variations in NTO-Al0.05 likely reflect overdoping effects, as they deviate from theoretical predictions for Al^3+^ substitution. Notably, examination of the β angles revealed that NTO-Al0.03 exhibited the largest value among all samples, indicating that optimal Al doping induces measurable lattice distortion in NTO. The potential impact of this structural modification on electrochemical performance requires further investigation in subsequent analyses.

The SEM images (Figure S4) display the surface microstructures of pristine NTO and Al-doped NTO samples (NTO-Al0.01, NTO-Al0.03, and NTO-Al0.05). Low-magnification images reveal irregular aggregates with randomly stacked morphologies, characteristics of conventional solid-state synthesis, and inherited precursor features. High-resolution observations show all samples possess nanorod-like structures several micrometers in length, though with slight variations in width and surface roughness, where the rods appear coated with fine particulate debris. Notably, while NTO-Al0.01 to NTO-Al0.03 maintain similar morphologies, with only marginal nanorod size reduction at higher doping levels, NTO-Al0.05 exhibits more irregular rods with non-uniform dimensions. This size refinement suggests Al doping may elevate oxide surface energy, slightly inhibiting crystal growth during calcination. The resultant smaller grain size and increased surface area could enhance electrochemical performance by shortening Na^+^ diffusion paths, reducing volume strain during (de)intercalation, and providing additional active sites.

**Figure 2:**
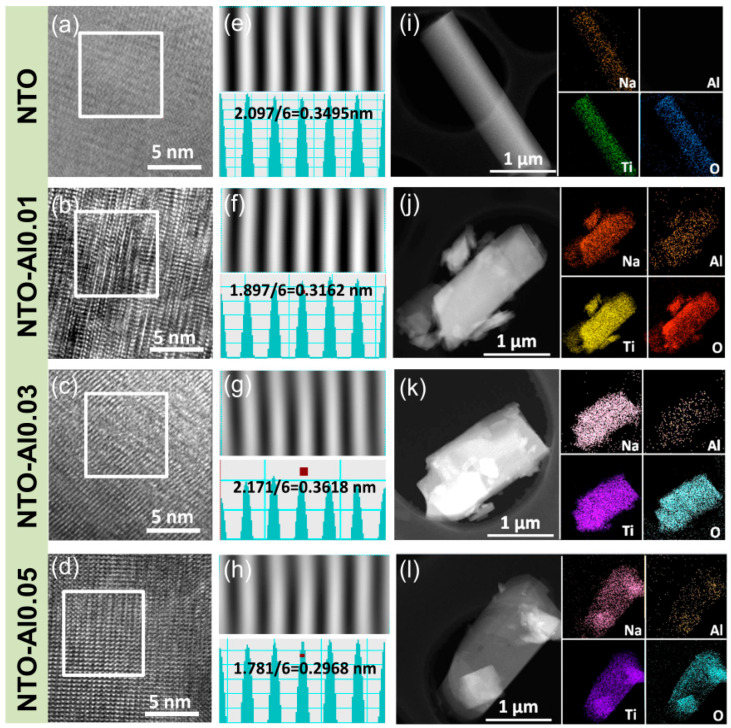
(**a**–**d**) HRTEM images of NTO-Alx (x = 0, 0.01, 0.03, 0.05) samples, (**e**–**h**) inverse FFT images of each sample and their corresponding line profiles, (**i**–**l**) STEM images and related EDX elemental maps for Na, O, Ti, and Al taken from the same zone of STEM images with the scale bar being 1 μm.

To further investigate the microstructure of the samples, Figure 2 presents TEM images of NTO and NTO-Alx particles. All samples exhibit rod-like particle structures with well-defined crystalline regions (Figure 2i–l). The HRTEM images (Figure 2a–d) reveal distinct lattice fringes, while the corresponding inverse fast Fourier transform (IFFT) images and line profiles (Figure 2e–h) provide accurate measurements of the d-spacings. For undoped NTO, the IFFT image shows an interplanar spacing of 0.3495 nm, matching the (110) plane of Na_2_Ti_3_O_7_ as calculated by Bragg’s law (2dsin θ = nλ) [36]. Similarly, the NTO-Al0.01 sample exhibits an average fringe spacing of 0.3162 nm, corresponding to the (111) plane of Na_2_Ti_3_O_7_, while the NTO-Al0.03 sample displays a spacing of 0.3618 nm, consistent with the (102) plane [37]. The NTO-Al0.05 sample shows a spacing of 0.2968 nm, aligning with the (003) plane [38]. Notably, the HRTEM images did not capture the (100) plane, which corresponds to the strongest XRD peak, due to the random orientation of the small regions analyzed in TEM and the challenges in resolving larger d-spacings in IFFT patterns, where reciprocal lattice points are more densely packed and harder to distinguish. Consequently, TEM analysis could not provide consistent comparisons of specific crystallographic planes across different doped samples. Finally, EDX mapping confirms the uniform distribution of Na, Ti, and O in all samples, along with homogeneous Al dispersion in the doped samples, indicating successful incorporation of Al into the Na_2_Ti_3_O_7_ crystal structure, in agreement with the XRD results.

**Figure 3:**
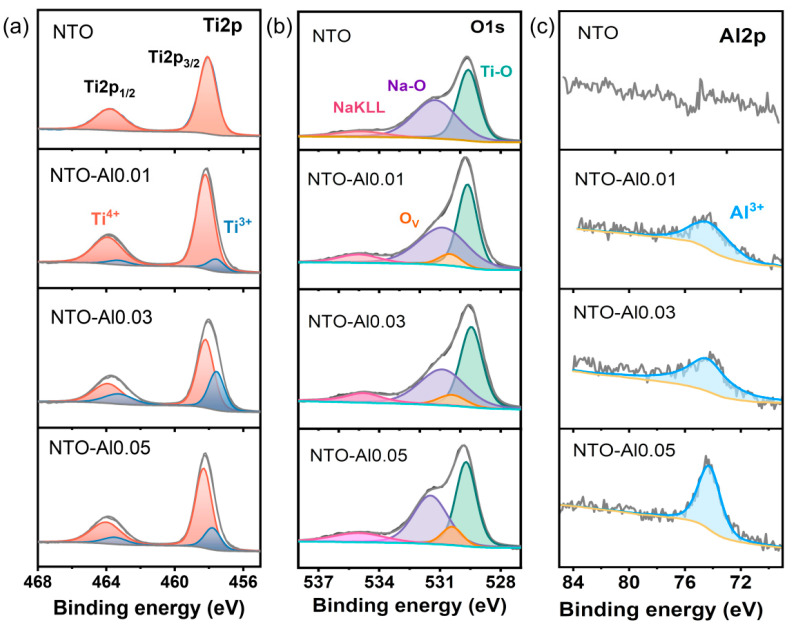
XPS spectra taken from the NTO-Alx (x = 0, 0.01, 0.03, and 0.05) samples of (**a**) Ti 2p spectra, (**b**) O 1s spectra, and (**c**) Al 2p spectra.

To gain deeper insights into the influence of Al doping on the chemical composition and electronic states of NTO materials, XPS analyses were conducted on both undoped and doped samples. High-resolution spectra of Ti2p, O1s, C1s, and Al2p were collected. As shown in Figure 3a, the Ti2p spectrum of pristine NTO exhibits two characteristic peaks at 458.1 eV (Ti2p_3/2_) and 463.8 eV (Ti2p_1/2_), with a spin–orbit splitting of 5.7 eV, confirming the dominant presence of Ti^4+^ [39]. In contrast, the Al-doped sample NTO-Al0.01 displays additional small peaks at lower binding energies (457.6 eV for Ti2p_3/2_ and 463.3 eV for Ti2p_1/2_), indicating partial reduction of Ti^4+^ to Ti^3+^ upon Al doping [40]. The Ti2p spectra of NTO-Al0.01 were thus deconvoluted into two components, with the lower-energy peaks assigned to Ti^3+^ [41]. Similarly, NTO-Al0.03 and NTO-Al0.05 also exhibit Ti^3+^ signatures, with NTO-Al0.03 showing the most pronounced Ti^3+^ contribution, suggesting a higher concentration of reduced Ti species in this sample. In the O1s region (Figure 3b), the partial reduction of Ti^4+^ induces a shift in oxygen vacancy-related peaks toward higher binding energies. The O1s spectrum of undoped NTO was fitted with three peaks at 529.6 eV (O-Ti bonds), 531.2 eV (O-Na bonds), and 534.9 eV (Na Auger peak). The doped samples exhibit an additional small peak at a slightly higher binding energy than the O-Ti peak, attributed to oxygen vacancies. According to the Kröger–Vink notation, the substitution of Ti^4+^ by lower-valent Al^3+^ in Na_2_Ti_3_O_7_ necessitates the formation of oxygen vacancies to maintain charge neutrality while preserving the original crystal structure. These oxygen vacancies increase electron density around Ti ions, lowering their binding energy and facilitating the generation of Ti^3+^. The presence of oxygen vacancies enhances carrier density and improves electrical conductivity [42], which is crucial for electrochemical performance. The Al 2p peak appears as a single symmetric peak, characteristic of Al^3+^ in oxides, with no observable spin–orbit splitting (Figure 3c) [43]. The intensity of the Al 2p peak increases with higher Al_2_O_3_ doping levels, confirming the incorporation of Al^3+^. However, while NTO-Al0.05 exhibits the highest Al^3+^ content, it does not show the highest Ti^3+^ or oxygen vacancy concentrations. Combined with XRD refinement results, this suggests that excessive Al doping may lead to unfavorable lattice distortions. In contrast, NTO-Al0.03 demonstrates an optimal balance, with the highest Ti^3+^ and oxygen vacancy concentrations, likely due to the compensation effect of Ti^3+^ (ionic radius: 0.67 Å) for the smaller Al^3+^ ions, mitigating lattice mismatch. In conclusion, controlled Al doping introduces beneficial Ti^3+^ and oxygen vacancies, significantly enhancing the electronic conductivity of NTO anodes. NTO-Al0.03 represents the optimal doping level, where the synergistic effects of Ti^3+^ and oxygen vacancies improve charge transport without inducing detrimental structural changes.

**Figure 4:**
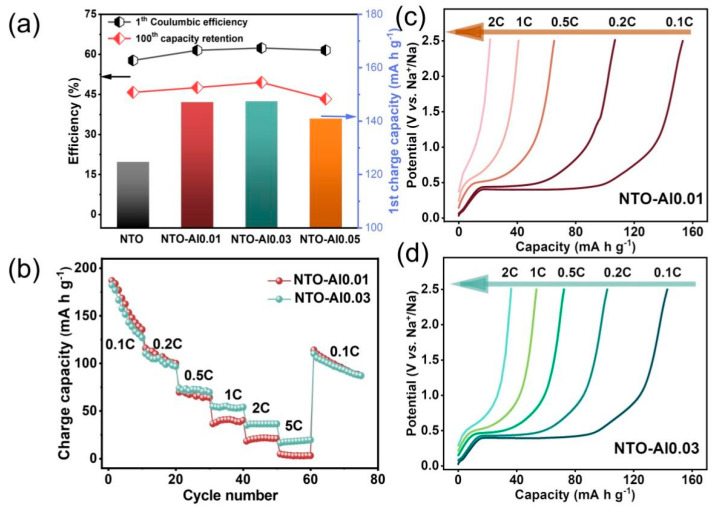
(**a**) Comparison chart of ICE, capacity retention rate, and first charge capacity; (**b**) rate performance of the NTO-Al0.01 and NTO-Al0.03 samples; the charge curves of (**c**) NTO-Al0.01 and (**d**) NTO-Al0.03.

Figure 4a demonstrates the electrochemical performance of Al-doped NTO samples at 0.5 C. The initial charge capacities of NTO-Al0.01 and NTO-Al0.03 reach 147.1 and 147.4 mA h g^−1^, respectively, slightly higher than NTO-Al0.05 (140.9 mA h g^−1^) and significantly exceeding pristine NTO (124.7 mA h g^−1^). This confirms that Al doping effectively increases active sites in NTO materials to enhance reversible capacity. Correspondingly, the initial Coulombic efficiency (ICE) improves from 57.7% for NTO to 61.5% (NTO-Al0.01), 62.4% (NTO-Al0.03), and 61.6% (NTO-Al0.05). After 100 cycles at 0.5 C, the capacity retention follows the order: NTO-Al0.03 (49.5%) > NTO-Al0.01 (47.6%) > NTO (45.8%) > NTO-Al0.05 (43.3%). Although all samples show relatively low cycling stability due to intrinsic limitations of NTO materials, NTO-Al0.03 maintains a superior discharge capacity of 72.9 mA h g^−1^ at the 100th cycle compared to 57.1 mA h g^−1^ for pristine NTO. Notably, NTO-Al0.05 shows slightly worse retention than undoped NTO, indicating that while Al doping inherently enhances stability, excessive doping (NTO-Al0.05) adversely affects cycling performance. Figure 4b presents rate performance of NTO-Al0.01 and NTO-Al0.03 (10 cycles per rate). While both demonstrate similar capacities below 0.5 C, NTO-Al0.03 exhibits significantly higher capacity at ≥1 C rates (e.g., 36.3 vs. 20.85 mA h g^−1^ at 2 C). As shown in Figure 4c,d, NTO-Al0.03 maintains distinct voltage plateaus even at 2C, whereas NTO-Al0.01 nearly loses this feature. When returning to 0.1C, both samples recover similar capacities, but NTO-Al0.03 shows a slight capacity increase during 5C cycling, while NTO-Al0.01 displays gradual degradation. The superior rate performance of NTO-Al0.03 correlates with its higher oxygen vacancy concentration (confirmed by XPS), which enhances electronic conductivity.

**Figure 5:**
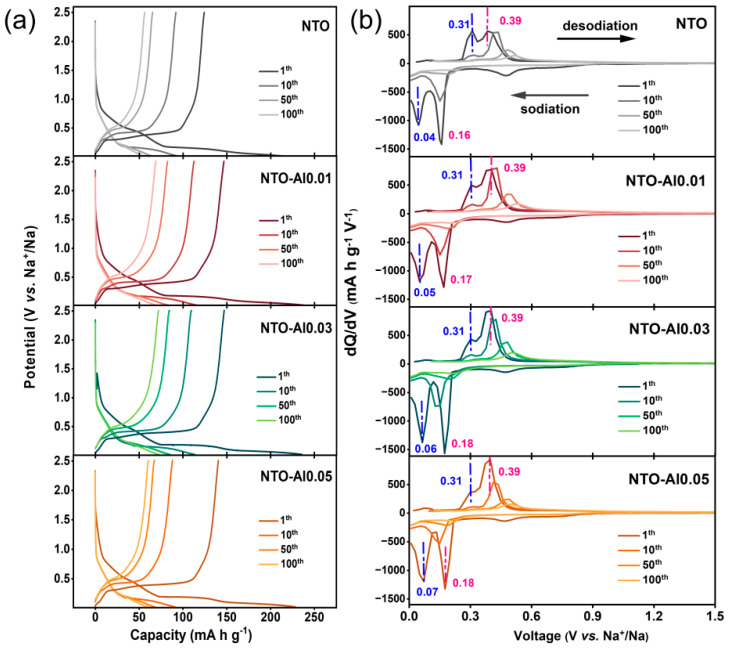
(**a**) Charge/discharge curves and (**b**) corresponding dQ/dV curves of NTO-Alx (x = 0, 0.01, 0.03, 0.05) samples at 88.5 mA g^−1^ current during 100 cycles.

Figure 5a shows the charge/discharge curves of different samples during cycling. All samples lose part of their voltage plateaus quickly in the first 10 cycles, then show slower capacity fading later. To understand their storage mechanisms better, we analyzed the differential capacity (dQ/dV) curves in Figure 5b. The reduction peaks (sodiation) are stronger than oxidation peaks (desodiation) for all samples, meaning Na_2_Ti_3_O_4_ (NTO) materials have faster reduction than oxidation. In the first cycle, besides the irreversible peak at 0.3–0.6 V (from electrolyte decomposition and SEI formation), all samples show two clear oxidation and reduction peaks. These come from two different Na storage sites in NTO: A low-voltage site (~0.31 V for oxidation) that works fast but stores less energy, and a high-voltage site (~0.39 V for oxidation) that stores more energy but works slower. In Al-doped samples, the high-voltage peak (~0.39 V) becomes much stronger than the low-voltage one (~0.31 V). This suggests Al doping helps Na ions move more easily in NTO. The peak positions stay almost the same for all samples, except small changes in the first-cycle reduction peak. With more Al doping, the reduction voltage shifts slightly higher. By the 10th cycle, the low-voltage reduction peak (~0.05 V) almost disappears, and its matching oxidation peak (~0.31 V) becomes very weak. The higher-voltage peaks (~0.17 V reduction and ~0.39 V oxidation) stay stronger, but NTO and NTO-Al0.05 show weaker peaks than NTO-Al0.01 and NTO-Al0.03. This means Al doping changes the NTO structure during cycling, affecting the Na^+^ deintercalation/intercalation behavior.

**Figure 6:**
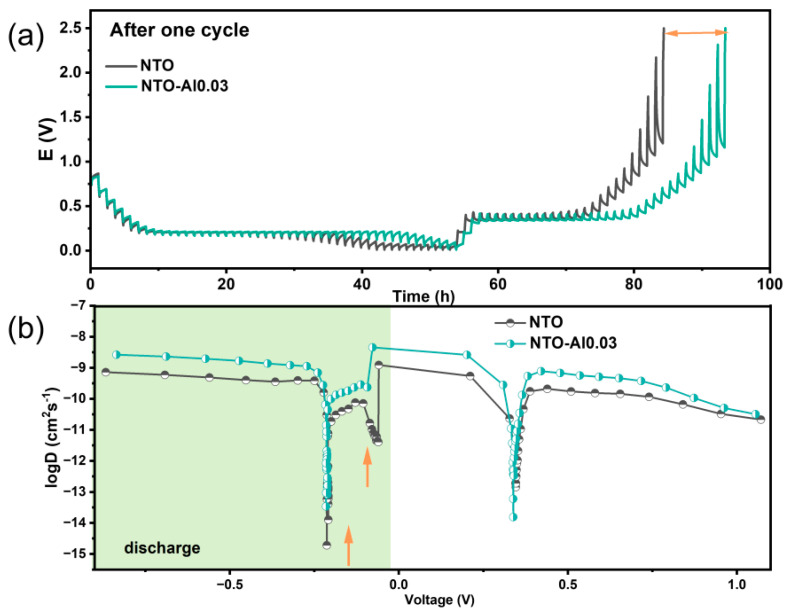
(**a**) GITT profiles and (**b**) calculated Na-ion diffusion coefficients during the two cycles at 0.2 C current of the NTO and NTO-Al0.03.

Figure 6a presents the potential response of electrodes during GITT measurements after one cycle at 0.2C. The potential variation during each relaxation period reflects the overpotential at different sodiation/desodiation stages. Notably, NTO-Al0.03 exhibits smaller overpotentials than pristine NTO at the same voltage, particularly in higher voltage ranges, indicating superior kinetic properties. The calculated Na⁺ diffusion coefficients (D_Na_) are shown in Figure 6b. Both electrodes initially show decreasing D_Na_ values during sodiation (above the plateau voltage), corresponding to the sloping region of discharge curves. As sodiation progresses, the material transitions from single-phase to two-phase coexistence, leading to varied diffusion behaviors. The sharp D_Na_ drop at plateau voltages occurs because Na^+^ must overcome repulsive charge gradients from pre-inserted ions for further diffusion. During desodiation, D_Na_ generally decreases with reduced Na content, except at the desodiation plateau.

Despite similar trends, NTO-Al0.03 demonstrates D_Na_ values at least one order of magnitude higher than NTO in non-plateau regions, attributed to Al doping-induced oxygen vacancies that facilitate faster Na^+^ migration. Notably, at the desodiation plateau, NTO-Al0.03 shows lower D_Na_, suggesting deeper and more reversible Na^+^ extraction, consistent with its higher charge capacity. Two distinct voltage regions (marked by arrows) in NTO exhibit particularly low D_Na_ during sodiation. Since D_Na_ depends not only on sodiation degree (e.g., vacancy concentration) but also phase transitions and lattice/electronic changes, these regions likely reflect unfavorable reduction reactions caused by poorer electronic conductivity in undoped samples, leading to irreversible Na⁺ trapping.

**Figure 7:**
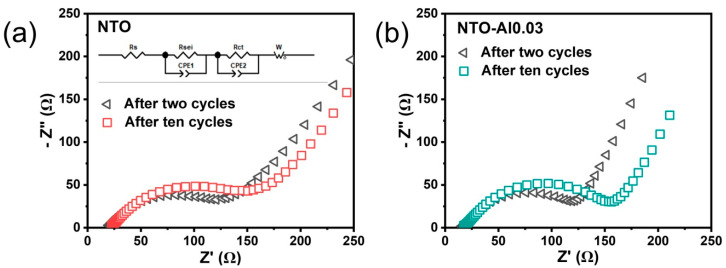
The Nyquist plots of (**a**) the NTO cells and (**b**) the NTO-Al0.03 cells after two cycles and ten cycles; inset figure is the equivalent circuit.

Electrochemical impedance spectroscopy (EIS) was used to further analyze the effect of Al doping on electrode impedance. Before EIS testing, all half-cells underwent complete charge/discharge cycles and reached equilibrium at their end-of-charge voltage. Figure 7a,b show the Nyquist plots of NTO and NTO-Al0.03 electrodes after two and ten cycles. The impedance spectra consist of overlapping semicircles in the high-to-medium frequency region and a straight line in the low-frequency region, which were analyzed using the simplified equivalent circuit model shown in the Figure 7a inset. The intercept at the Z_re_ axis in the high-frequency region corresponds to the ohmic resistance (R_s_), representing the resistance of the electrolyte and electrodes [44,45]. The semicircles in the high-to-medium-frequency range represent the SEI film resistance (R_SEI_) and charge transfer resistance (R_ct_), while the constant phase element (CPE) in parallel represents the double-layer capacitance and SEI film capacitance. The sloping line in the low-frequency region indicates the Warburg impedance (Z_w_), which is related to sodium ion diffusion within NTO particles. The plots clearly show that both NTO and NTO-Al0.03 samples exhibit increased resistance from 2 to 10 cycles.

For more detailed comparison, Table S4 lists the specific resistance values obtained from equivalent circuit fitting. The R_s_ values of these samples show little difference, indicating that Al doping and cycling numbers did not significantly affect the electrolyte–electrode interaction, as expected from using identical electrode formulations and electrolytes. The R_sei_ and R_ct_ values show slight variations, and NTO exhibits higher resistance (100.0 Ω) than NTO-Al0.03 (85.5 Ω) after two cycles, but after ten cycles, the resistance of the pristine NTO shows a slight decrease (81.0 Ω), whereas that of NTO-Al0.03 exhibits a moderate increase (113.2 Ω), possibly due to increased interface resistance caused by slightly reduced particle size in NTO-Al0.03. The Z_w_ values constitute a major portion of the total resistance (R_total_), demonstrating that ion diffusion is a critical step in the entire (de)intercalation process. The smaller Z_w_ values of NTO-Al0.03 at both two (74.8 Ω) and ten (88.7 Ω) cycles, along with its much slower resistance increase compared to NTO, indicate that Al doping effectively enhances ion and electron transfer kinetics at the electrode. These results confirm that appropriate Al doping reduces electrode resistance and represents an effective approach to improve the electrochemical activity of NTO materials.

**Figure 8:**
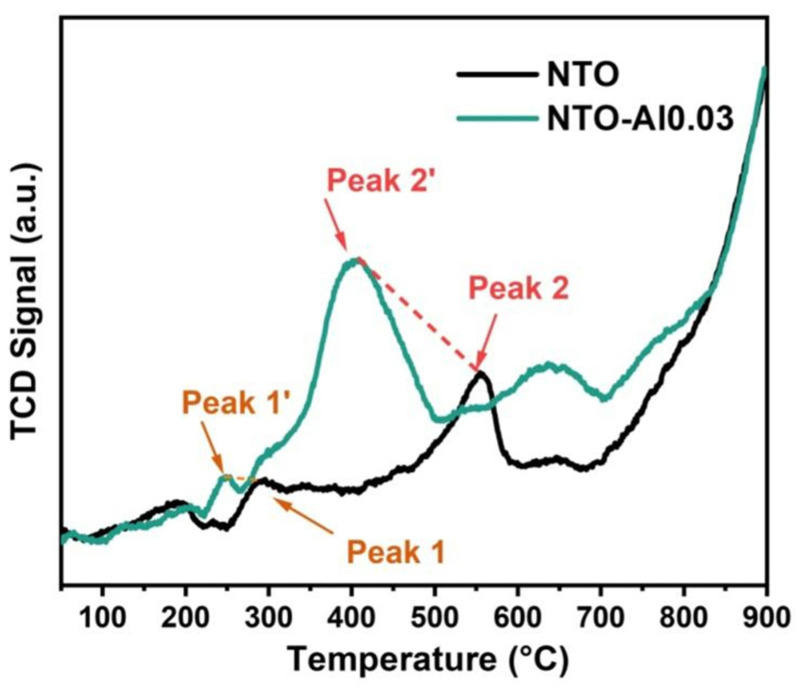
The H_2_-TPR diagrams of NTO and NTO-Al0.03 samples.

For NTO electrodes, sodium insertion/extraction follows a phase transition mechanism where multiple phases coexist during discharge/charge processes, with the electrode performance closely related to the Ti^3+^/Ti^4+^ redox characteristics. A H_2_-TPR analysis was conducted to examine the reduction capability from Ti^4+^ to Ti^3+^ in both NTO and NTO-Al0.03. As shown in Figure 8, Peak1 corresponds to surface oxygen while Peak2 represents lattice oxygen. After doping, both peaks shift to lower temperatures (Peak1 from 294 °C to 248 °C; Peak2 from 554 °C to 410 °C), indicating that Al doping activates lattice oxygen and enhances its participation in reactions. The lower reduction temperatures of NTO-Al0.03 demonstrate that Al doping facilitates the reduction of Ti^4+^ to Ti^3+^, thereby improving the redox reaction kinetics of the electrode.

## 4. Conclusions

This work proposed an Al- and Ca-doped NTO electrode materials using a one-step solid-state synthesis method. We successfully prepared Al-doped NTO-Alx (x = 0.01, 0.03, 0.05) and Ca-doped NTO-Cay (y = 0.02, 0.04, 0.06, 0.08, and 0.10) samples, with comprehensive characterization confirming effective Al or Ca incorporation into the NTO lattice. The Ca-doped samples did not significantly improve the first-cycle reversible capacity of NTO but enhanced cycling stability. After 100 cycles, optimized NTO-Ca0.06 only delivered a capacity of 70.9 mA h g^−1^. The optimal NTO-Al0.03 sample demonstrated enhanced electrochemical performance, delivering a higher initial charge capacity (147.4 vs. 124.7 mA h g⁻^1^ for pristine NTO at 0.5C), improved cycling stability (49.5% capacity retention after 100 cycles), and better rate capability (36.3 mA h g^−1^ at 2 C). Detailed mechanistic investigations revealed that Al doping facilitated faster Na^+^ diffusion through the formation of oxygen vacancies and reduced charge-transfer resistance while simultaneously stabilizing the host structure during cycling. These findings demonstrate that controlled Al doping can effectively improve both the capacity and kinetics of NTO anode materials for sodium-ion battery applications. However, while Al doping effectively enhances electronic conductivity, it fails to sufficiently suppress the structural degradation in NTO anodes, and NTO-Alx exhibited an unsatisfied cycling stability. Future optimization should employ integrated approaches combining interface engineering (e.g., carbon coating/artificial SEI), electrolyte modification, and nanostructural design to concurrently address interfacial instability and mechanical failure.

## Data Availability

Data are contained within the article.

## Supplementary Materials

The following supporting information can be downloaded at https://www.mdpi.com/article/10.3390/nano15120885/s1, Figure S1: XRD patterns of NTO and doping samples; Figure S2: Cycle performance of NTO with (a) Al doping samples, (b) Ca doping samples, (c) cycle performance comparison of all samples; Figure S3: (a) Rate performance of NTO and doping samples, (b) rate capacity comparison of all samples; Figure S4: SEM images of NTO-Alx (x = 0, 0.01, 0.03, and 0.05) samples; Table S1: Elemental analysis based on ICP results and theoretical results; Table S2: The ratio of Raman peak intensities of Na_2_Ti_3_O_7_ and doping samples; Table S3: The refinement results from XRD patterns of Na_2_Ti_3_O_7_ and doping samples; Table S4: The impedance values of NTO and NTO-Al0.03 samples based on equivalent circuit fitting.
