# Supplementary material for: Improving Na2Ti3O7 Anode Performance in Sodium-Ion Batteries via a Al Doping
Source: Nanomaterials (Basel). 2025 Jun 8;15(12):885. doi: 10.3390/nano15120885 (PMC12195616; doi:10.3390/nano15120885)
Supplement: Supplementary file 1 [file nanomaterials-15-00885-s001.zip › nanomaterials-3640103-supplementary.pdf]

Supporting Information

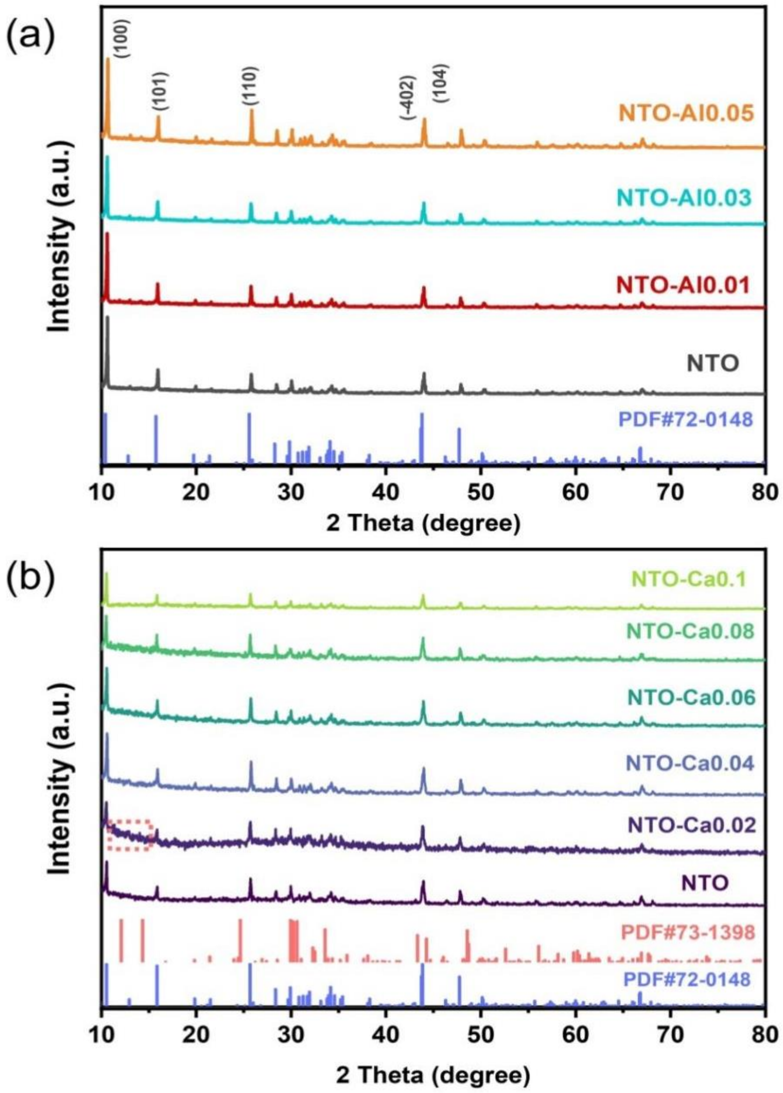

Figure S1. XRD patterns of NTO and doping samples.

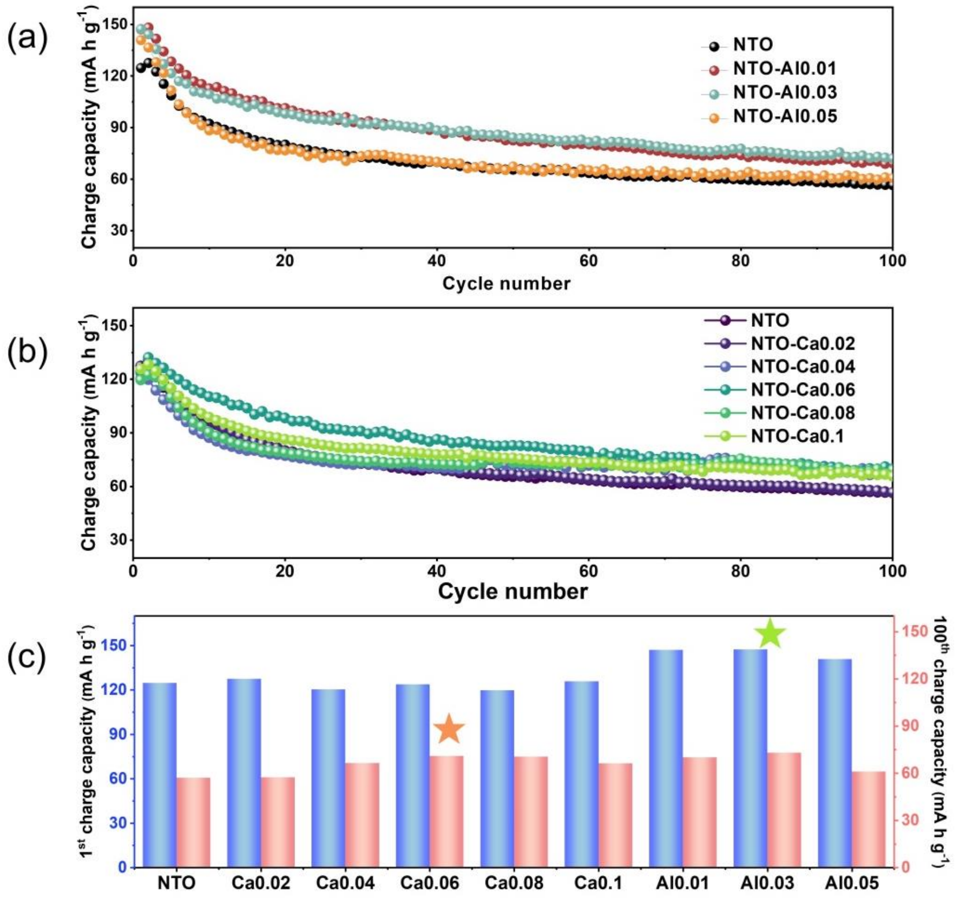

Figure S2. Cycle performance of NTO with (a) Al doping samples, (b) Ca doping samples, (c) cycle performance comparison of all samples.

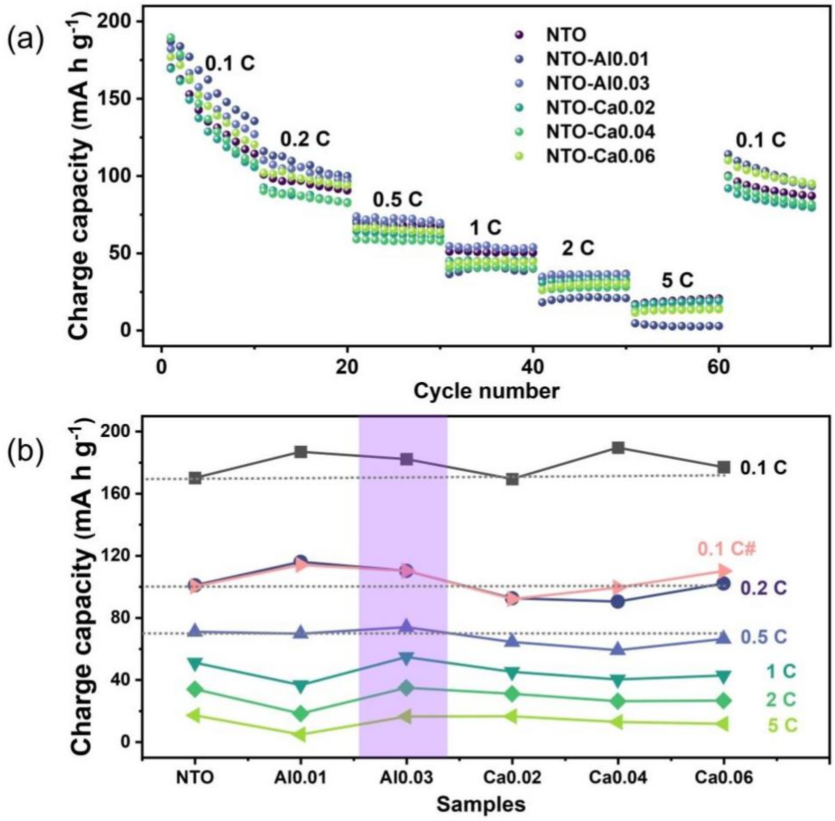

Figure S3. (a) Rate performance of NTO and doping samples, (b) rate capacity comparison of all samples.

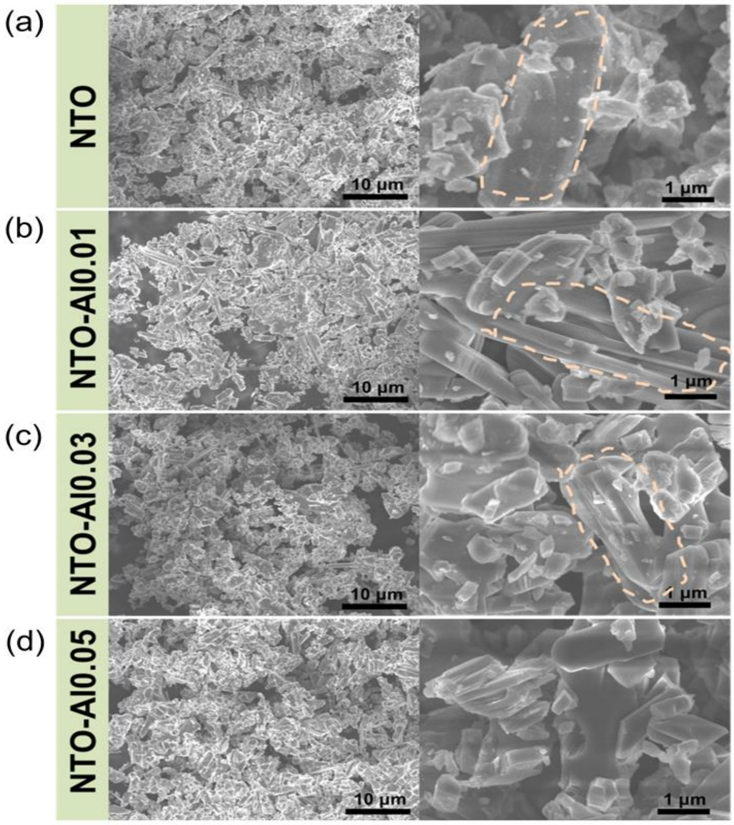

Figure S4. SEM images of NTO-Alx (x=0, 0.01, 0.03, and 0.05) samples.

Table S1. Elemental analysis based on ICP results and theoretical results.

Samples	ICP Ti content (%)	ICP Al content (%)	ICP Ti/Al	Ti/Al (Ti site doping)
NTO-Al0.01	47.04	0.09	294.58	299.25
NTO-Al0.03	47.33	0.26	102.60	99.25
NTO-Al0.05	46.98	0.44	60.18	59.25

Table S2. The ratio of Raman peak intensities of $Na_2Ti_3O_7$ and doping samples.

Samples	81 cm^{-1}	299 cm^{-1}	845 cm^{-1}	881 cm^{-1}
NTO	0.47	1	0.57	0.84
NTO-Al0.01	0.43	1	0.45	0.85
NTO-Al0.03	0.44	1	0.48	0.80
NTO-Al0.05	0.50	1	0.59	0.90

Table S3. The refinement results from XRD patterns of $Na_2Ti_3O_7$ and doping samples.

Samples	*a* (Å)	*b* (Å)	*c* (Å)	*β* (°)	*V* ($Å^3$)
NTO	8.5628(3)	3.8006(8)	9.1240(6)	101.60(4)	290.86(9)
NTO-Al0.01	8.5621(7)	3.8007(2)	9.1238(4)	101.60(7)	290.84(0)
NTO-Al0.03	8.5630(6)	3.8005(4)	9.1234(1)	101.61(2)	290.83(9)
NTO-Al0.05	8.5657(6)	3.8015(3)	9.1261(9)	101.60(4)	291.10(2)

Table S4. The impedance values of NTO and NTO-Al0.03 samples based on equivalent circuit fitting.

Samples	R_s (Ω)	R_{sei}&R_{ct} (Ω)	Z_w (Ω)	R_{total} (Ω)
NTO (2nd)	17.2	100.0	119.3	236.5
NTO (10th)	19.5	81.0	199.6	300.1
NTO-Al0.03 (2nd)	15.0	85.5	74.8	175.3
NTO-Al0.03 (10th)	17.2	113.2	88.7	219.1
